# Medical Science in China

**Published:** 1884-11

**Authors:** 


### Medical Science in China.

In view of the impending war in China there is a demand for foreign, especially American medical talent. Like most other arts and sciences in that strange land, medicine and surgery suffered an arrest of development centuries ago, and have since made no progress. Only the simplest surgical operations are undertaken, and it is quite probable that in the event of a war as many will die from the lack of proper surgical skill as will fall by the bullets of the French. A few months since the writer visited the Smithsonian Institute in Washington, in company with a person who has spent several years in China. We were much interested in examining the collection of Chinese medicines, and listening to an account of their methods of practice, an occupation which was, to say the least, entertaining if not instructive.

The medical notions of the Chinese have many points of resemblance with those of Europe in the middle ages, being a mixture of truths derived from experience with many absurd speculations and superstitions. Like our modern refined disciples of Hahnemann, the Chinese physician believes in the specific action of drugs, and employs remedies which rival, in nastiness, the homœopathic tinctures of bed-bugs, pediculus capitis and psorinum syphiliticum. The Pen-tsan-kong-mu, which corresponds, in a measure, to our National Dispensatory, was published in the sixteenth century, and is still the standard authority. It contains a list of about 1,900 crude drugs. These drugs are seldom given singly, but are combined in complicated formulæ which surpass, in length, those of the celebrated Dr. Brown-Sequard. The Chinese Dispensatory contains over 12,000 of these formulæ, so that there is, at least, an ample field for selection.

Only the rudest chemical knowledge is apparent. Inorganic substances, when used, are in their native mineral state, and the general form for administering all drugs is in powder or decoction. In the selection of remedies, there are many illustrations of the mediæval doctrine of signatures, that nature has indicated by certain peculiarities of form, color, or otherwise, the proper use of a drug. Ginseng root is most highly prized, as it presents a rude resemblance to the human form. Marvelous invigorating properties are attributed to it. It is given to the sick as a restorative, to the well as a preventive; in fact, it is the Chinese quinine. As further illustrations of this law of signatures, we may mention the use of red coral to arrest hemorrhage, hedgehog skin for cutaneous diseases, and tigers' blood as a remedy for timidity and debility. The consumption of drugs is enormous; many medicines are habitually taken by those in perfect health in the belief that they prolong life and prevent disease.

Some of their remedies are really efficacious. Aconite is used to reduce fever, but only after some method of preparation has been employed to remove its poisonous properties. Ginger is used as a stomachic tonic and for headaches. Musk and camphor are favorite remedies; these are given combined, in the form of a bolus about half an inch in diameter, and are excellent nervous stimulants for those who have gullets sufficiently large to swallow them. Japonica root, resembling, in its action, squill, is used as a diuretic and expectorant. Fowls' gizzards are given for dyspepsia, it is said, with excellent results, while red rose leaves are regarded as a specific for asthma.

The tonics used by the Chinese will interest us only on account of their absurdity. Among them may be mentioned the gall-stones found in the gall-bladders of cattle; a variety of glues made out of asses' hide, cowhide and deer horns; tigers' blood and bones, dried toads, and dried human placenta. Among other curious remedies may be mentioned, caterpillars, for bronchitis; snake skin, dried and powdered, for cutaneous diseases, especially leprosy; cuttle-fish, for cancer; oyster shell, for deafness, and maggots for the delirium of fever.

We are not surprised that with this pharmacopœia the Chinese physician is not a great success. Most foreign medical men attain wealth and distinction, and are now welcomed in many of the larger cities of the Empire.

